# Supplementary material for: Biomarkers of Clot Activation and Degradation and Risk of Future Major Cardiovascular Events in Acute Exacerbation of COPD: A Cohort Sub-Study in a Randomized Trial Population
Source: Biomedicines. 2022 Aug 19;10(8):2011. doi: 10.3390/biomedicines10082011 (PMC9405886; doi:10.3390/biomedicines10082011)
Supplement: Supplementary file 1 [file biomedicines-10-02011-s001.zip › supplementary document s1.pdf]

ADDITIONAL FILE 2:

Missing values:

CRP was missing completely at random and was handled with multiple imputation.

FEV_1% of predicted could be missing at random or not missing at random. For the cases where FEV_1 (liter) was present, this was calculated based on the GLI references in SAS. When FEV_1 was measured at 30-day follow-up, but not baseline (n=8), stochastic regression was performed yielding the best possible estimate for FEV_1 at baseline. The remainder of missing data was handled with multiple imputation, as described below. Further, worst-case/best-case imputation was performed on FEV_1% of predicted as sensitivity analyses.

Multiple imputation was performed in the SMCFCS package, for each substantial model, with 100 imputations, 20 iterations and a rejection limit of 2000 and the following auxiliary variables: HsCRP (dichotomized above/below 20), oxygen supplement, treatment with non-invasive ventilation during admission.
